# Multidrug-Resistant Commensal and Infection-Causing *Staphylococcus* spp. Isolated from Companion Animals in the Valencia Region

**DOI:** 10.3390/vetsci11020054

**Published:** 2024-01-26

**Authors:** Ana Marco-Fuertes, Clara Marin, Concepción Gimeno-Cardona, Violeta Artal-Muñoz, Santiago Vega, Laura Montoro-Dasi

**Affiliations:** 1 Departamento de Producción y Sanidad Animal, Salud Pública Veterinaria y Ciencia y Tecnología de los Alimentos, Facultad de Veterinaria, Instituto de Ciencias Biomédicas, Universidad Cardenal Herrera-CEU, CEU Universities, Calle Santiago Ramón y Cajal 20, Alfara del Patriarca, 46115 Valencia, Spain; ana.marcofuertes@uchceu.es (A.M.-F.); clara.marin@uchceu.es (C.M.); 2 Servicio de Microbiología, Consorcio Hospital General Universitario de Valencia, Facultad de Medicina, Universidad de Valencia, 46014 Valencia, Spain; concepcion.gimeno@uv.es; 3 Servicio de Microbiología, Consorcio Hospital General Universitario de Valencia, 46014 Valencia, Spain; artal_vio@gva.es

**Keywords:** companion animals, antimicrobial resistance, *Staphylococcus*, methicillin-resistant *Staphylococcus aureus*, methicillin-resistant *Staphylococcus pseudintermedius*

## Abstract

**Simple Summary:**

The increase in microorganisms resistant to antimicrobials poses a growing risk to the effectiveness of medical treatments, both in humans and animals. This surveillance is essential to understand and address the magnitude of the problem and its impact on public health. Therefore, it is crucial to monitor antimicrobial resistance not only in human medicine but also in veterinary medicine. Companion animals, in particular, play a significant role as they live in close contact with their owners, potentially facilitating the transmission of these antimicrobial resistance between people and animals. Thus, this study aimed to evaluate the epidemiological situation of antimicrobial resistance in dogs and cats to the opportunistic pathogen *Staphylococcus* spp. The main results showed a high prevalence of antimicrobial resistance in the study population (healthy and diseased dogs and cats), even to the last resort of antibiotics in human medicine, which poses a threat to global public health.

**Abstract:**

The emergence of antimicrobial resistance (AMR) and multidrug resistance (MDR) among microorganisms to commonly used antibiotics is a growing concern in both human and veterinary medicine. Companion animals play a significant role in the epidemiology of AMR, as their population is continuously increasing, posing a risk of disseminating AMR, particularly to strains of public health importance, such as methicillin-resistant *Staphylococcus* strains. Thus, this study aimed to investigate the prevalence of AMR and MDR in commensal and infection-causing *Staphylococcus* spp. in dogs and cats in Valencia region. For this purpose, 271 samples were taken from veterinary centers to assess antimicrobial susceptibility against 20 antibiotics, including some of the most important antibiotics for the treatment of *Staphylococcus* infections, including the five last resort antibiotics in this list. Of all the samples, 187 *Staphylococcus* spp. strains were recovered from asymptomatic and skin-diseased dogs and cats, of which *S. pseudintermedius* (≈60%) was more prevalent in dogs, while *S. felis* (≈50%) was more prevalent in cats. In the overall analysis of the isolates, AMR was observed for all antibiotics tested, including those crucial in human medicine. Furthermore, over 70% and 30% of the strains in dogs and cats, respectively, exhibited MDR. This study highlights the significance of monitoring the trends in AMR and MDR among companion animals. The potential contribution of these animals to the dissemination of AMR and its resistance genes to humans, other animals, and their shared environment underscores the necessity for adopting a One Health approach.

## 1. Introduction

In an ever-changing society, companion animals are increasingly living in close contact with their owners in their homes, but they also share public spaces, such as parks or beaches, with animals (domestic or wild) and other people, including the elderly, children, or immunosuppressed patients [1]. In fact, the population of companion animals continues to grow in importance and members in European households (230 million dogs and cats) [2,3].

In this context, new challenges have arisen because zoonotic pathogens, multi-resistant bacteria, and their resistance genes can be spread and acquired through the environment they share [4,5]. Among these hazards, AMR and the emergence of multidrug resistance (MDR) are one of the most important problems facing public health, according to the World Health Organisation (WHO) [6]. In fact, the study conducted in 2019 revealed that there were 1.27 million deaths directly caused by AMR per year [7].

Over time, the trends in AMR have evolved, conditioned by the implementation of new regulations focused on controlling past overuse of antibiotics in both human and animal health [8,9]. As a result of these efforts, including surveillance and monitoring programmes and the European Medicines Agency (EMA) categorisation for the responsible use of antibiotics in veterinary medicine [10], the use of antibiotics in animal production has decreased. However, companion animals have not been included in all of these control measures. Within this framework, the European Union (EU) is developing the European Antimicrobial Resistance Surveillance network in veterinary medicine (EARS-Vet), which aims to monitor AMR in the main pathogens affecting companion animals (dogs and cats) together with food-producing animals [11,12], to complement the existing European AMR Surveillance Network (EARS-Net) in human medicine [13] in order to achieve a global view of this problem under the “One Health” strategy. Nevertheless, there is a lack of studies evaluating the epidemiological situation of AMR in companion animals, although it is necessary to establish a starting point [5].

To study the epidemiology of AMR, *Escherichia coli* has been the main sentinel bacterium used, due to its ability to acquire and transfer AMR genes, as it is a commensal bacterium that is part of the microbiota of animals and humans [14,15]. However, it is necessary to research the AMR problem from more perspectives. For this reason, Gram-positive bacteria belonging to the family *Staphylococcaceae*, which are considered part of the commensal microbiota of the skin and mucous membranes of animals and humans, are also used as an indicator of resistance [16]. Within this family, two groups are distinguished: coagulase-positive *Staphylococcus* (CoPS) [17] and coagulase-negative *Staphylococcus* (CoNS) [18]. Most CoPS are opportunistic pathogens and cause the majority of infections at the dermal level in humans and animals [19]. They are known to acquire resistant genes to a large extent, so treatment options against these bacteria are limited, making infections difficult to treat, especially those caused by methicillin-resistant *Staphylococcus aureus* (MRSA) and methicillin-resistant *Staphylococcus pseudintermedius* (MRSP) [20,21,22]. Regarding CoNS, although they are not as common in causing infections, they are widely recognised as commensal organisms of the skin microbiota and opportunistic pathogens of humans and animals [18,23]. Therefore, the aim of this study was to establish the presence of commensal vs. infection-causing *Staphylococcus* spp. and the epidemiological situation of their AMR and MDR in companion animals (dogs and cats) in the Valencia region.

## 2. Materials and Methods

### 2.1. Experimental Design

The animal sampling procedure was evaluated and authorised by the Animal Ethics Committees of UCH-CEU University (permit Nº. CEEA 22/04).

Veterinary hospitals (VHs) and clinics (VCs) located across the Valencia region were invited to take part voluntarily in this study. Out of these, eight veterinary centers voluntarily consented to collaborate: three large reference VHs, handling cases from the entire Valencia region, and five VCs, spread throughout the Valencia region.

### 2.2. Epidemiological Data Collection

First, an epidemiological questionnaire for each animal was completed, together with the informed consent signed by the owners (Supplementary Materials, Part A), in order to classify the animals depending on their epidemiologic characteristics and be able to evaluate their effect on the appearance of AMR and MDR. The information collected was related to the origin of the animals (VH vs. VC) and general information such as sex and age. Regarding the age of the animals, a general classification described by Marco-Fuertes et al. (2023) was used to group dogs and cats [24]. Moreover, whether they cohabit with other animals and the clinical data of each animal were included (chronic diseases, daily medication, and antibiotic treatment received). Finally, the data regarding dogs and cats were analysed individually.

### 2.3. Sample Collection

Dogs and cats were sampled between October 2022 and June 2023 in order to isolate *Staphylococcus* spp. To isolate commensal *Staphylococcus*, a single swab (Cary–Blair sterile transport swabs, DELTALAB, Barcelona, Spain) was first introduced in the nasal cavity and then in the auricular cavity, approximately 3 cm [25,26], from healthy asymptomatic dogs and cats. Before taking the samples, the veterinarians performed a clinical examination in which they assessed the animals’ vital signs to confirm that they were within normal ranges, thus classifying them as asymptomatic healthy animals. For the isolation of infection-causing *Staphylococcus,* animals with active skin infections were sampled by taking a Cary–Blair sterile transport swabs, which were then introduced into skin-infected wounds. After collecting the samples, all of them were preserved in Cary–Blair transport medium and transported under refrigeration at ≤4 °C to the microbiology laboratory within 24 h of sampling to the Faculty of Veterinary Sciences of the University CEU Cardenal Herrera.

### 2.4. Staphylococcus Isolation

A pre-enrichment in buffered peptone water (BPW; Scharlau, Barcelona, Spain), at a ratio of 1:10 *v*/*v*, of the sample swabs collected were carried out, followed by an incubation at 37 ± 1 °C for 24 h. After that, the suspension was streaked onto the non-specific agar Columbia CNA agar with 5% sheep blood, Improved II (BD, Becton Dickinson, Madrid, Spain), and incubated at 37 ± 1 °C for 24–48 h. The plates were examined at 24 and 48 h, and the suspected colonies, matching the typical morphology of *Staphylococcus* spp. in blood agar and the positive result of the catalase test, were identified using a MALDI-TOF MS Biotyper System (Bruker Daltonics, Madrid, Spain) at the Microbiology Service of the *Consorcio Hospital General Universitario de Valencia*.

### 2.5. Antimicrobial Susceptibility Testing

Antimicrobial susceptibility was evaluated using the minimum inhibition concentration (MIC) assay (Thermo Scientific™ Sensititre™ Plates, Madrid, Spain) using a panel of 20 antibiotics applied in human medicine and of importance in public health (Table 1) [11]. In addition, the plate presented two D-test wells. D-test wells combine two antibiotics (clindamycin (CLI) and erythromycin (ERY)), indicating whether the strain tested has inducible resistance to CLI in the presence of ERY and may therefore lead to therapeutic failure. The interpretation was carried according to the Spanish Society of Infectious Diseases and Clinical Microbiology (*SEIMC*, from its Spanish acronym *Sociedad Española de Enfermedades Infecciones y Microbiología Clínica*) [27].

Each bacterial strain was cultured and revived on nutrient agar and then incubated at 37 ± 1 °C for 24 h. After the incubation period, the colonies were transferred into 5 mL of sterile demineralised water (T3339; Thermo Fisher Scientific™, Madrid, Spain). The suspension of each bacterium was mixed and adjusted to achieve a 0.5 McFarland using a nephelometer (Sensititre™ Nephelometer, Thermo Fisher Scientific™, Madrid, Spain). Subsequently, 10 μL of the suspension were introduced into a vial containing 11 mL of Mueller–Hinton broth (T3462; Thermo Fisher Scientific™, Madrid, Spain) and mixed. From this suspension, 50 μL of the vial contents were transferred into each Sensititre plate well (GPALL1F, Thermo Fisher Scientific™, Madrid, Spain). Then, the plates were incubated at 37 ± 1 °C for 24 h and manually examined using a Sensititre Vizion (Thermo Scientific™ Sensititre™ Vizion™ Digital MIC Viewing System, Thermo Fisher Scientific, Madrid, Spain).

Finally, the results were interpreted following the guidelines established by the European Committee on Antimicrobial Susceptibility Testing (EUCAST) in its last report (14th ed., 2024) [29]. Methicillin-resistant *Staphylococcus* (MRS) strains were studied by monitoring the AMR observed against oxacillin + 2% NaCl for *S. pseudintermedius* (the antibiotic used for screening MRSP strains) and against cefoxitin for *S. aureus* and CoNS (the antibiotic used for screening MRSA and MR-CoNS strains). However, some MIC values for these two antibiotics for screening MRSP and methicillin-resistant coagulase-negative *Staphylococcus* (MR-CoNS) are not currently available in the EUCAST, so the Clinical and Laboratory Standards Institute (CLSI) recommendations specified in M100 [30] and VET01 [31] were followed. Moreover, MDR was characterized as the acquired resistance to at least one agent in three or more antimicrobial classes [32]. Finally, according to the EARS-Vet [11], the following detailed results are those obtained for *S. aureus* and *S. pseudintermedius*, while the rest of the information detailed on the AMR observed for each of the isolated species can be found in the Supplementary Materials, Table S1.

### 2.6. Statistical Analysis

A generalised linear model (GLM) with a probit link function, assuming a binomial distribution, was applied to the data to examine the influence of external factors on AMR and MDR patterns. This analysis aimed to determine associations with categorical variables such as animal origin, sex, cohabitation with other animals (and number of animals, if applicable), relationship with animals outside the household, and clinical information regarding chronic diseases, daily medication, and previous antibiotic treatments. In addition, a probit link function GLM was performed, assuming a binomial distribution for AMR patterns in *Staphylococcus* spp. from dogs and cats, for the microbiological results. A *p*-value of ≤0.05 was considered indicative of a statistically significant difference. Data were presented as the least squares means ± standard error of least squares means. Statistical analyses were performed using the R software (version 4.3.1) packages EMMs [33], car [34], and multicompView [35].

## 3. Results

### 3.1. Epidemiological Data

Among the sampled population (*n* = 271), there were 152 dogs and 119 cats. Regarding the samples’ origin, 43.9% of the samples were taken in VHs (79/152 and 40/119, dogs and cats, respectively), and 56.1% of the samples were collected in VCs (73/152 and 70/119, dogs and cats, respectively).

**Figure 1:**
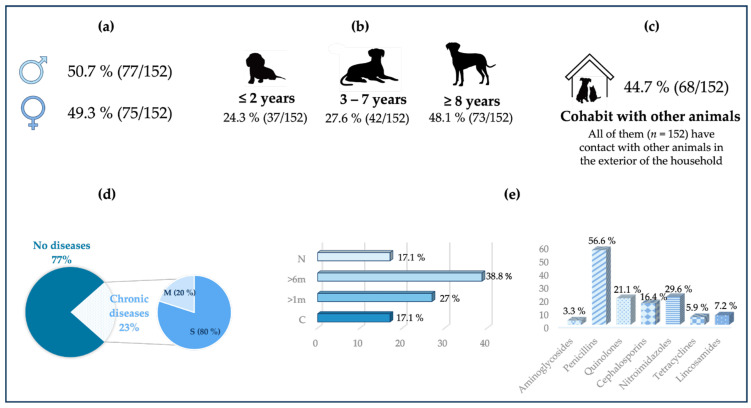
Epidemiological data for all the dogs sampled. (**a**) Distribution of the study population by sex. (**b**) Distribution of the study population by age. (**c**) The relationship of the animals in the study population with other animals. *n*: total number of animals. (**d**) Whether the animals of the study population present any disease and of which type. M: musculoskeletal. S: systemic. (**e**) Previous antibiotic therapy (**left** graph) and antibiotics administered at some point in their lives (**right** graph). N: never. >6 m: in the last six months. >1 m: in the last month. C: currently.

**Figure 2:**
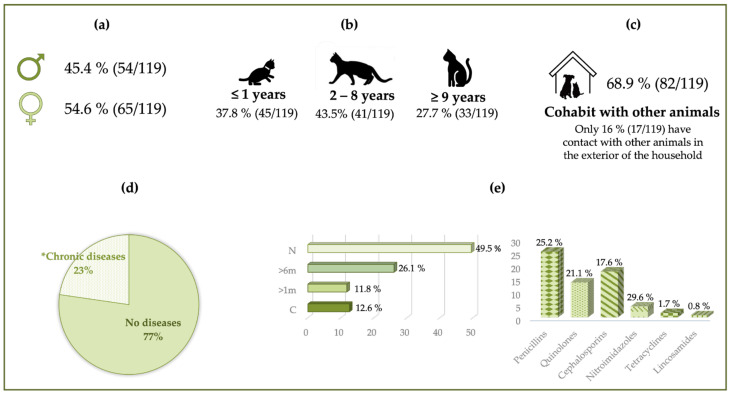
Epidemiological data for all the cats sampled. (**a**) Distribution of the study population by sex. (**b**) Distribution of the study population by age. (**c**) The relationship of the animals in the study population with other animals. (**d**) Whether the animals of the study population present any disease. *Chronic diseases: all of them are classified as systemic diseases. (**e**) Previous antibiotic therapy (**left** graph) and antibiotics administered at some point in their lives (**right** graph). N: never. >6 m: in the last six months. >1 m: in the last month. C: currently.

As reported in the Materials and Methods section, an epidemiological survey for each animal was collected. Figure 1 compiles all the information collected in the questionnaire regarding the dog samples, while Figure 2 compiles all the information related to the cat samples.

### 3.2. Staphylococcus Prevalence

The prevalence of *Staphylococcus* spp. from all the samples taken, including dogs and cats, was 69% (187/271).

From all the canine samples collected, the prevalence of *Staphylococcus* was 74.3% (113/152), of which 74.3% (84/113) and 25.7% (29/113) were commensal and infection-causing *Staphylococcus*, respectively.

Regarding the samples collected from cats, the prevalence of this bacterium was 62.2% (74/119). About the prevalence of this bacterium according to the type of sample, 87.8% (65/74) were commensal *Staphylococcus*, and 12.2 % (9/74) were infection-causing *Staphylococcus*. All *Staphylococcus* species isolated from dogs and cats and the type of sample from which they are derived are detailed in Table 2.

### 3.3. Antimicrobial Susceptibility in Staphylococcus Strains

#### 3.3.1. Methicillin Resistance

**Figure 3:**
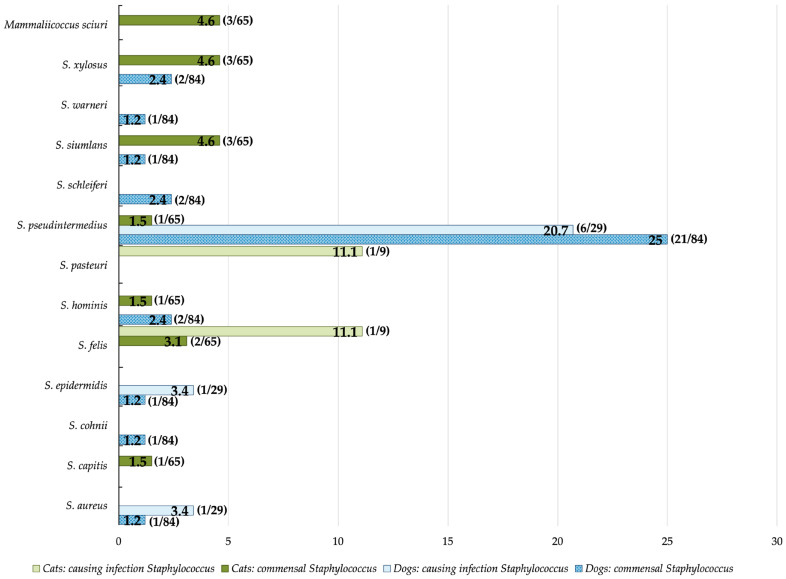
Percentage and number of methicillin-resistant *Staphylococcus* strains classified by *Staphylococcus* species according to their observed phenotypic resistance to oxacillin + 2% NaCl and cefoxitin (the two antibiotics used to screen methicillin-resistant *Staphylococcus* strains).

In all the *Staphylococcus* strains, 30.5% (57/187) were MRS, of which 71.9% (41/57) belonged to dogs and 28.1% (16/57) belonged to cats. All the results regarding the sampled animals (dogs or cats), the strain species, and the strain’s origin (commensal or infection) are represented in Figure 3.

#### 3.3.2. Dogs

Of all the commensal *Staphylococcus* isolated from healthy asymptomatic dogs, 95.2% (80/84) showed AMR to at least 1 of the 20 antibiotics studied, and 72.6% (61/84) were considered MDR, while only 4.8% (4/84) of the strains were sensitive to all the antibiotics studied. Regarding the AMR of these strains for the different antibiotic groups evaluated, they are ordered from the highest to the lowest percentage: 59.5% for amphenicols, 56% for macrolides, 54.4% for penicillins, 47.6% for glycylcyclines, 46.4% for tetracyclines, 42.9% for lincosamides, 40.5% for cephalosporins, 28.6% for oxazolidinones, 28.6% for quinolones, 11.9% for aminoglycosides, 10.7% for folate inhibitor pathway, 9.5% for streptogramins, 7.1% for ansamycins, 3.6% for glycopeptides, 3.6% for nitrofurans, and 2.4% for lipopeptides. In the D-test we performed, 35.7% (30/84) of the strains were positive.

On the other hand, only 10.3% (3/29) of the infection-causing *Staphylococcus* isolated from animals with active skin infections were sensitive to all the antibiotics tested, while 89.7% (26/29) of the strains presented AMR and 55.2% (16/29) were MDR. The AMR in each antibiotic group were (from the highest percentage to the lowest percentage) as follows: 51.7% for macrolides, 49.4% for penicillins, 44.8% for amphenicols, 44.8% for lincosamides, 41.4% for tetracyclines, 26.4% for quinolones, 24.1% for cephalosporins, 20.7% for aminoglycosides, 17.2% for folate inhibitor pathway, 10.3% for oxazolidinones, 6.9% for glycylcyclines, 6.9% for streptogramins, and 3.4% for glycopeptides, and no resistance was found for the lipopeptides, nitrofurans, and ansamycins. Concerning the D-test results, 41.4% (12/29) of the strains tested positive.

In addition, no correlation was observed between the clinical data collected in the questionnaire and the appearance of AMR and MDR (*p*-value > 0.05).

Of all the strains isolated, *S. aureus* and *S. pseudintermedius* were the main strains with importance in public health. Therefore, their AMR levels are detailed in Table 3 and Table 4.

#### 3.3.3. Cats

Regarding all the commensal *Staphylococcus* strains isolated from healthy asymptomatic cats, 21.5% (14/65) were susceptible to the 20 antibiotics studied, while 75.4% (49/65) showed AMR to at least one of the antibiotics studied, and 32.3% (21/65) were MDR. In addition, the AMR observed of all cat strains studied against each group of antibiotics, ordered from the highest to the lowest percentage, was: 32.3% for macrolides, 27.7% for lincosamides, 21.5% for amphenicols, 21.5% for tetracyclines, 25.1% for penicillins, 21.5% for cephalosporins, 15.4% for ansamycins, 13.8% for streptogramins, 11.8% for quinolones, 9.2% for aminoglycosides, 7.7% for lipopeptides, 6.2% for nitrofurans, and 4.6% for the folate inhibitor pathway, glycopeptides, glycylcyclines, and oxazolidinones. In the results observed from the D-test, 13.8% (9/65) tested positive.

For all the infection-causing *Staphylococcus* isolated from cats with active skin infections, 11.1% (1/9) were sensitive to all the antibiotics studied, while 88.9% (8/9) were AMR, and 55.6% (5/9) were MDR. Ordered from the highest to the lowest percentage, the AMR of all the strains in each antibiotic group was: 51.9% for penicillins, 44.4% for amphenicols, 44.4% for lincosamides, 44.4% for macrolides, 44.4% for tetracyclines, 37% for quinolones, 22.2% for cephalosporins, and 11.1% for the glycopeptides, lipopeptides, nitrofurans, ansamycins, and streptogramins. None of the isolated strains showed resistance to aminoglycosides, the folate inhibitor pathway, glycylcyclines, or oxazolidinones. Regarding the D-test performed in the infection-causing *Staphylococcus* strains, 22.2% (2/9) were positive. Furthermore, no relationship was observed between the clinical data collected in the questionnaire and the manifestation of AMR and MDR *(p*-value > 0.05).

As mentioned for dogs, *S. aureus* and *S. pseudintermedius* were the main strains with importance in public health. Therefore, their AMR levels are shown in Table 5 and Table 6. However, no infection-causing *S. pseudintermedius* was isolated from cats with active skin infections.

Overall, the AMR trends did not follow any pattern, as 126 different AMR patterns were observed in the 187 *Staphylococcus* spp. strain isolates in this study. Of all the AMR patterns, 62.7% (79/126) belonged to *Staphylococcus* spp. isolated from dogs, and 37.3% (47/126) belonged to *Staphylococcus* spp. isolated from cats. The most common AMR pattern was observed in the penicillin group alone in dogs (4%, 5/126) and in cats (3.2%, 4/126), followed by the macrolides group alone in cats (3.2%, 4/126). All AMR patterns are attached in the Supplementary Materials, Table S2.

## 4. Discussion

The emergence of AMR and MDR strains in companion animals represents a new challenge for global public health, which must be addressed through a One Health strategy [36,37]. This is not only crucial due to therapeutic failures in veterinary medicine but also in human medicine. Studies have shown that these strains can circulate in the environment and be transmitted from animals to humans and vice versa [38]. Therefore, it is essential to assess the presence of this resistance in both commensal and pathogenic bacteria.

In this study, a new genus of the *Staphylococceae* family has been studied, as, due to new phylogenomic studies of this family, some *Staphylococcus* have been relocated to other genera [39]. The study that proposed this taxonomic reassignment was published relatively recently, and therefore MALDI-TOF and other biochemical analyses continue to identify these bacteria as *Staphylococcus*, as in the case of *S. sciuri* (former CoNS) that now belongs to the genus *Mammaliicoccus sciuri* [39,40]. Nevertheless, the implications that *M. sciuri* has on public health remains the same, as it is considered one of the most ancient species in natural history capable of carrying virulence and AMR genes similar to those identified in other *Staphylococcal* species. However, the scientific community has increasingly focused on *M. sciuri*, mainly because this species is believed to be the most likely evolutionary reservoir of the *mecA* gene, which has subsequently spread to *S. aureus* and other *Staphylococcus* species [41].

The overall observed prevalence of *Staphylococcus* spp. and *Mammaliicoccus sciuri* in our study (69%) aligns with that reported in previous studies [42,43]. In line with this, it has also been seen that both healthy and diseased companion animals harbor both CoPS and CoNS, although certain species showed a stronger association with each animal species [43]. In this study, CoPS, such as *S. pseudintermedius*, were more frequently isolated from dogs, while CoNS, including *S. felis*, were more commonly isolated from cats, as reported previously [42,43,44,45]. This finding is significant, given the widespread observation of methicillin resistance not only in CoPS but also in CoNS, as both groups are now acknowledged as important pathogens [43,46,47]. Nevertheless, the pathogenic potential of CoNS has not been as well studied as the virulence factors involved in CoPS, although it has been recognised that they cause some important diseases, such as endocarditis or urinary tract infections in at-risk populations, and that they are important reservoirs of AMR genes [48,49]. Thus, it is essential to monitor the commensal microbiota of animals, and not only the most “relevant” bacterial species, to detect the risks of human exposure to animal species [50].

Regarding the AMR obtained, similar rates of AMR and MDR were found in both commensal and infection-causing *Staphylococcus* isolates from dogs and cats. However, upon comparison, high levels of AMR were observed in dogs, consistent with findings published in other studies [51]. One hypothesis that could explain these results is that dogs have more contact with other animals and humans, given their daily walks and shared public spaces. In contrast, cats typically live indoors, as observed in our study, where only 16% of cats went outdoors.

Antimicrobial agents effective in treating infections caused by these organisms are limited, particularly for *Staphylococcus* strains that exhibit MDR, including MRS [52]. In this study, the highest prevalence of MRS was observed in dog *S. pseudintermedius* (37% and 33.3% in commensal and infection-causing isolates, respectively). Similar results were reported in a study conducted in Tennessee (USA), where 30.8% of the isolates were MRSP [52].

Overall, the antibiotics with the highest percentages of AMR were those in the penicillins group (almost 50% in dogs and cats), chloramphenicol (≈50% in dogs and 25% in cats), erythromycin (≈47% for both species), clindamycin (≈40% for both), and tetracycline (≈40% for both). Similar results have been observed in other studies conducted in the Iberian Peninsula [51] and Canada [53]. However, lower AMR profiles were observed in another study conducted in the USA [52]. In some Scandinavian countries, the main resistance observed was to penicillins in different proportions, with 65% in Denmark [54], ≈70% in Finland [55], 14% in Norway [56], and 19.5% in Sweden [57]. These data have been published in their latest national reports on antimicrobial resistance, but the difference in these results may be due to the *Staphylococcus* species studied in each programme, as only *S. pseudintermedius* were studied in Denmark, *S. aureus* and *S. pseudintermedius* in Finland, and only *S. felis* in Norway and Sweden (as only MRSP and MRSA strains of these two species were evaluated in these countries). For the other antibiotics studied, their results varied between countries, but in all countries, the AMR rates were lower than in this study. This variation may be attributed to the geographical area or choice of antibiotics for treating infections influenced by regional legislation [58]. In addition, it is important to highlight that the observed AMR to the penicillins group was significantly higher in dogs than in cats. These findings could be linked to the administration of penicillins in our study population, as it emerged as the most commonly prescribed antibiotic group for both dogs and cats, with dogs receiving it twice as frequently (56.6%) as cats (25.2%).

In terms of the AMR observed in some of the most important public health species, due to their pathogenic capacity and ability to harbor resistance genes, both *S. pseudintermedius* and *S. aureus* showed similar patterns in the commensal and infection-causing isolates from dogs, with the highest AMR observed against penicillins (≈80%), chloramphenicol (≈57%), erythromycin (≈56%), tetracycline (≈50%), and clindamycin (≈48%), in accordance with Lord et al. (2022) [59]. Although there are not many isolates of *S. aureus* in our study, the results align with those observed by other authors in different geographical areas, such as Nepal [60], Italy [61], India [62], Bangladesh [63], or the USA [64], highlighting the concerning emergence of AMR in these strains, posing a threat to public health. The high levels of AMR to chloramphenicol are particularly concerning, not only for *S. pseudintermedius* (almost 70%) but also for *S. aureus* (more than 80%), given its usefulness for the treatment of MRS infections [59]. Something similar happens with erythromycin and clindamycin, two antibiotics of choice in the treatment of MRSA and MRSP [65,66]. Therefore, the D-test was performed in this study. The observed results were slightly higher in dogs than in cats. In both cases, this inducible phenotype was observed to a greater extent in infection-causing strains, which may lead to treatment failure due to the development of constitutive resistance [60]. Thus, all strains with a positive D-test should be reported as being resistant to clindamycin [67].

In the new WHO medically important antimicrobial list, quinolones belonged to the highest priority critically important antimicrobials (HPCIAs), antibiotics that should only be used in veterinary medicine when all others have failed [28]. Overall, the AMR of quinolones was around 30%, varying from one *Staphylococcus* species to another, as seen in different studies [68,69,70]. In particular, levofloxacin and moxifloxacin had the highest AMR in the quinolones group in both dog and cat *S. aureus* and *S. pseudintermedius*, regardless of whether they were commensal or infection-causing strains. Even though these quinolones are not authorised for veterinary use in the EU but only for human use [71], these antimicrobials are experiencing an increase in both human [72,73] and animal [61,74] strains worldwide.

Finally, regarding the antibiotics that are only authorised for human use and not intended for animals, commonly known as last resort antibiotics, five of them were tested in this study: vancomycin, tigecycline, daptomycin, lincomycin, and nitrofurantoin. The results on tigecycline resistance are particularly alarming, as high rates have been observed in dog isolates, especially in commensal *S. pseudintermedius* (46.3%). Similar results have been observed in human medicine, ranging from 5.6% [75,76] to almost 30% [77], and up to 88% in CoNS of other animal species, such as turkeys [78]. This high acquired AMR for this antibiotic represents a major public health concern, as it is one of the newest last resort antibiotics used to treat MDR infections caused by *Staphylococcus* strains [79,80]. On the other hand, all cat isolates were susceptible to tigecycline, as reported in other studies in isolates from cats and other animal species [81,82,83], as well as humans [83,84].

Another of these antibiotics with high AMR found in this study was lincomycin, with the highest resistance observed in commensal *S. aureus* (83.3%) and *S. pseudintermedius* (25.9%), as well as in infection-causing *S. aureus* (33.3%) isolated from dogs. Most of the cases reporting lincomycin resistance in *Staphylococcus* strains are from intensive care units [85] and hospitals [84,86], so the expected results would have been that, as in other studies carried out in different countries such as Portugal [72,78], Brazil [68], Italy [73], and China [79], no resistance to this antibiotic would be observed.

Regarding vancomycin, it is often used for severe infections caused by MRSA and MR-CoNS strains, among other complicated infections [87]. In the present study, of all the *Staphylococcus* spp. isolated, only eight showed AMR to vancomycin, four from dogs and four from cats. The findings in other studies regarding this antibiotic suggest that its AMR is rare in companion animals [70,77,88]. However, higher VAN-resistant *S. aureus* strains have been previously reported in bovine mastitis [89] and human medicine in hospitals [84]. In addition, a low range of AMR has also been seen against daptomycin (4%), rifampicin (8%), and quinupristin/dalfopristin (10%). These antibiotics are reserved to treat fastidious and MDR infections, mainly caused by Gram-positive bacteria [90,91]. In fact, DAP is mostly reserved to treat VAN-resistant infections [92]. In the case of RIF and QUD, these are antibiotics that previously also belonged to the same category as DAP but have now been relocated to the CIA and HIA categories, respectively. Some studies show higher percentages of AMR to DAP and RIF from human isolates [84]. Different AMR rates have been observed in companion animals [93,94], as reported by Burke et al. (2023) in a 10-year study, where only one *S. schleiferi* and one *S*. *pseudintermedius* isolated from dogs were resistant to RIF, while all cat strains were susceptible [69]. The same has been reported for DAP by Bellato et al. (2022) but with a higher AMR to RIF (12.5%) [82].

In contrast to the previous results mentioned, it is relevant to highlight that the AMR to NIT (around 5% for all the strains) in this study was among the lowest observed. In previous WHO and EMA classifications, this antibiotic was placed in the least important category [10]. However, in the latest WHO report, this antibiotic has been moved to the list of those not authorised for animal use [28]. Therefore, despite the limited knowledge on the mechanism of action of this antibiotic, it represents a potential tool in the fight against antimicrobial resistance, and further studies on this molecule are needed [95].

## 5. Conclusions

In conclusion, the results obtained in this study highlight the importance of following the new WHO categorisation when prescribing antibiotics for companion animals, as the highest resistance observed in this study is against the first treatments of choice for infection-causing *Staphylococcus* (amphenicols, macrolides, lincosamides, and tetracyclines). Nevertheless, no significant statistical differences were observed among epidemiological clusters. This is particularly concerning since AMR and MDR seem to be extensively disseminated, even in cases where animals have not undergone prior antibiotic treatments, including HPCIAs, and are not authorised antibiotics for animal use. These findings underscore the need to control companion animals as potential reservoirs and transmitters of resistance to both humans and the environment, following a One Health strategy. Moreover, further in-depth epidemiological studies of the transmission of AMR between companion animals and humans are needed to establish adequate control tools.

## Figures and Tables

**Table 1:** Antibiotics, World Health Organisation classification, and their concentrations included in the Sensititre plate for Gram-positive bacteria GPALL1F (Thermo Scientific™ Sensititre™, Madrid, Spain).

Antibiotic Group	Antibiotic	Abbreviation	WHO	Concentration
**Aminoglycosides**	Gentamicin	GEN	CIA	2–16 μg/mL
**Amphenicols**	Chloramphenicol	CHL	HIA	2–16 μg/mL
**Cephalosporins**	Cefoxitin ^1^	CXI	HIA	6 μg/mL
**Folate inhibitor pathway**	Trimethoprim/ sulfamethoxazole	TRS	HIA	1/19–8/152 μg/mL
**Glycopeptides**	Vancomycin	VAN	NA	0.25–32 μg/mL
**Glycylcyclines**	Tigecycline	TIG	NA	0.03–0.5 μg/mL
**Lincosamides**	Clindamycin	CLI	HIA	0.5–2 μg/mL
**Lipopeptides**	Daptomycin	DAP	NA	0.5–4 μg/mL
**Macrolides**	Erythromycin	ERY	CIA	0.25–4 μg/mL
**Nitrofurans**	Nitrofurantoin	NIT	NA	32–64 μg/mL
**Oxazolidinones**	Linezolid	LIN	NA	1–8 μg/mL
**Penicillins**	Ampicillin	AMP	HIA	0.25–8 μg/mL
	Oxacillin + 2% NaCl ^1^	OXA+	HIA	0.25–4 μg/mL
	Penicillin	PEN	HIA	0.06–8 μg/mL
**Quinolones**	Levofloxacin (FQ)	LEV	HPCIA	0,25–4 μg/mL
	Ciprofloxacin (FQ)	CIP	HPCIA	1–2 μg/mL
	Moxifloxacin (FQ)	MOX	HPCIA	0.25–4 μg/mL
**Tetracyclines**	Tetracycline	TET	HIA	2–16 μg/mL
**Ansamycins**	Rifampicin	RIF	CIA	0.5–4 μg/mL
**Streptogramins**	Quinupristin/ dalfopristin	QUD	HIA	0.5–4 μg/mL
**D-test**	Erythromycin (E) + clindamycin (C)	DT		4 μg/mL (E) + 0.5 μg/mL (C)

FQ: fluoroquinolone. ^1^: cefoxitin and oxacillin + 2% NaCl are two antibiotics used to screen methicillin-resistant *Staphylococcus* strains. WHO: World Health Organisation (this column indicates the last update of the classification of medically important antimicrobials authorised by the WHO for human and animal use in order to protect public health, updated in 2023 [28]). HIA: highly important antimicrobial. CIA: critically important antimicrobial. HPCIA: highest priority critical important antimicrobial. NA: not authorised for animal use.

**Table 2:** Prevalence of *Staphylococcus* species isolated from commensal mucosa and active skin infection samples from dogs and cats identified using a MALDI-TOF MS Biotyper System (Bruker Daltonics, Madrid, Spain).

	Type of Sample	Prevalence of *Staphylococcus* by Class	*Staphylococcus* Species	*n* and (%) Prevalence of Each Species
**Dog**	Commensal mucosa	CoPS—79.8%	*S. aureus*	6 (7.1)
			*S. pseudintermedius*	54 (64.2)
			*S. schleiferi*	7 (8.3)
		CoNS—20.2%	*S. cohnii*	1 (1.2)
			*S. epidermidis*	4 (4.8)
			*S. haemolyticus*	1 (1.2)
			*S. hominis*	3 (3.6)
			*S. sciuri* ^1^	2 (2.4)
			*S. simulans*	2 (2.4)
			*S. warneri*	2 (2.4)
			*S. xylosus*	2 (2.4)
	Active skin infection	CoPS—82.8%	*S. aureus*	4 (13.8)
			*S. pseudintermedius*	18 (62.2)
			*S. schleiferi*	2 (6.9)
		CoNS—17.2%	*S. canis*	1 (3.4)
			*S. chromogenes*	1 (3.4)
			*S. epidermidis*	2 (6.9)
			*S. felis*	1 (3.4)
**Cat**	Commensal mucosa	CoPS—16.9%	*S. aureus*	6 (9.2)
			*S. pseudintermedius*	3 (4.6)
			*S. schleiferi*	2 (3.1)
		CoNS—83.1%	*S. capitis*	2 (3.1)
			*S. epidermidis*	2 (3.1)
			*S. felis*	32 (49.2)
			*S. hominis*	1 (1.5)
			*S. pettenkoferi*	2 (3.1)
			*S. saprophyticus*	1 (1.5)
			*S. sciuri* ^1^	4 (6.2)
			*S. simulans*	6 (9.2)
			*S. xylosus*	4 (6.2)
	Active skin infection	CoPS—11.1%	*S. aureus*	1 (11.1)
		CoNS—88.9%	*S. epidermidis*	1 (11.1)
			*S. felis*	5 (55.6)
			*S. hominis*	1 (11.1)
			*S. pasteuri*	1 (11.1)

CoPS: coagulase-positive *Staphylococcus*. CoNS: coagulase-negative *Staphylococcus. n*: number of isolated strains. ^1^: *S. sciuri* is still identified as such in all identification databases but now belongs to a new genus due to new phylogenomic studies named *Mammaliicoccus sciuri*.

**Table 3:** Antimicrobial resistance in commensal and infection-causing *Staphylococcus aureus* isolated from healthy dogs and dogs with an active skin infection.

AB Group	AB	% AMR/AB in Commensal *S. aureus*	% AMR/AB in Infection-Causing *S. aureus*
**Aminoglycosides**	GEN	16.7 ^a,b^ (1/6) ± 15.2	0 ^a^ (0/4) ± 0
**Amphenicols**	CHL	83.3 ^c^ (5/6) ± 15.2	75 ^b^ (3/4) ± 21.7
**Cephalosporins**	CXI	16.7 ^a,b^ (1/6) ± 15.2	25 ^a,b^ (1/4) ± 21.7
**Folate inhibitor pathway**	TRS	0 ^b^ (0/6) ± 0	0 ^a^ (0/4) ± 0
**Glycopeptides**	VAN	50 ^a,c^ (3/6) ± 20.4	0 ^a^ (0/4) ± 0
**Glycylcyclines**	TIG	16.7 ^a,b^ (1/6) ± 15.2	25 ^a,b^ (1/4) ± 21.7
**Lincosamides**	CLI	50 ^a,c^ (3/6) ± 20.4	75 ^b^ (3/4) ± 21.7
**Lipopeptides**	DAP	0 ^b^ (0/6) ± 0	0 ^a^ (0/4) ± 0
**Macrolides**	ERY	83.3 ^c^ (5/6) ± 15.2	25 ^a,b^ (1/4) ± 25
**Nitrofurans**	NIT	0 ^b^ (0/6) ± 0	0 ^a^ (0/4) ± 0
**Oxazolidinones**	LIN	83.3 ^c^ (5/6) ± 15.2	25 ^a,b^ (1/4) ± 21.7
**Penicillins**	AMP	50 ^a,c^ (3/6) ± 20.4	50 ^a,b^ (2/4) ± 25
	PEN	83.3 ^c^ (5/6) ± 15.2	75 ^b^ (3/4) ± 21.7
**Quinolones**	LEV	16.7 ^a,b^ (1/6) ± 15.2	0 ^a^ (0/4) ± 0
	CIP	0 ^b^ (0/6) ± 0	0 ^a^ (0/4) ± 0
	MOX	16.7 ^a,b^ (1/6) ± 15.2	0 ^a^ (0/4) ± 0
**Tetracyclines**	TET	33.3 ^a,b^ (2/6) ± 19.2	25 ^a,b^ (1/4) ± 21.7
**Ansamycins**	RIF	16.7 ^a,b^ (1/6) ± 15.2	0 ^a^ (0/4) ± 0
**Streptogramins**	QUD	0 ^b^ (0/6) ± 0	25 ^a,b^ (1/4) ± 21.7

AB: antibiotic. AMR: antimicrobial resistance. GEN: gentamicin. CHL: chloramphenicol. CXI: cefoxitin. TRA: trimethoprim–sulfamethoxazole. TIG: tigecycline. CLI: clindamycin. DAP: daptomycin. ERY: erythromycin. NIT: nitrofurantoin. LIN: linezolid. AMP: ampicillin. penicillin. LEV: levofloxacin. CIP: ciprofloxacin. MOX: marbofloxacin. TET: tetracycline. RIF: rifampicin. QUD: quinupristin/dalfopristin. ^a–c^: the different superscripts in each column denote statistically significant variations (*p*-value ≤ 0.05) in the observed resistance to the antibiotics examined. ±: standard error.

**Table 4:** Antimicrobial resistance in commensal and infection-causing *Staphylococcus pseudintermedius* isolated from healthy dogs and dogs with an active skin infection.

AB Group	AB	% AMR/AB in Commensal *S. pseudintermedius*	% AMR/AB in Infection-Causing *S. pseudintermedius*
**Aminoglycosides**	GEN	11.1 ^a^ (10/54) ± 4.3	27.8 ^a,b,c,d^ (5/18) ± 10.6
**Amphenicols**	CHL	68.5 ^e,f^ (37/54) ± 6.3	50 ^a,c,e^ (9/18) ± 11.8
**Folate inhibitor pathway**	TRS	13 ^a,i^ (7/54) ± 4.6	22.2 ^b,c,d^ (4/18) ± 9.8
**Glycopeptides**	VAN	0 ^g^ (0/54) ± 0	5.6 ^b,g^ (1/18) ± 5.4
**Glycylcyclines**	TIG	46.3 ^c,d^ (25/54) ± 6.8	5.6 ^b,g^ (1/18) ± 5.4
**Lincosamides**	CLI	46.3 ^c,d^ (25/54) ± 6.8	50 ^a,c,e^ (9/18) ± 11.8
**Lipopeptides**	DAP	1.9 ^g,h^ (1/54) ± 1.8	0 ^g^ (0/18) ± 0
**Macrolides**	ERY	57.4 ^c,e^ (31/54) ± 6.7	55.6 ^a,e,f^ (10/18) ± 11.7
**Nitrofurans**	NIT	3.7 ^a,g,h^ (2/54) ± 2.6	0 ^g^ (0/18) ± 0
**Oxazolidinones**	LIN	25.9 ^i,j^ (14/54) ± 6	11.1 ^b,d,g^ (2/18) ± 7.4
**Penicillins**	AMP	44.4 ^b,c,d^ (27/54) ± 6.8	66.6 ^e,f^ (12/18) ± 11.1
	OXA+	37 ^b,d,j^ (21/54) ± 6.6	33.3 ^a,c,d^ (6/18) ± 11.1
	PEN	77.8 ^f^ (41/54) ± 5.7	83.3 ^f^ (15/18) ± 8.8
**Quinolones**	LEV	42.6 ^b,c,d,j^ (23/54) ± 6.7	44.4 ^a,c,e^ (8/18) ± 11.7
	CIP	0 ^g^ (0/54) ± 0	38.9 ^a,c,e^ (7/18) ± 11.5
	MOX	42.6 ^b,c,d,j^ (23/54) ± 6.7	33.3 ^a,c,d^ (6/18) ± 11.1
**Tetracyclines**	TET	51.9 ^c,d,e^ (28/54) ± 6.8	50 ^a,c,e^ (9/18) ± 11.8
**Ansamycins**	RIF	3.7 ^a,g,h^ (2/54) ± 2.6	0 ^g^ (0/18) ± 0
**Streptogramins**	QUD	7.4 ^a,h^ (3/54) ± 3.6	5.6 ^b,g^ (1/18) ± 5.4

AB: antibiotic. AMR: antimicrobial resistance. GEN: gentamicin. CHL: chloramphenicol. TRA: trimethoprim–sulfamethoxazole. TIG: tigecycline. CLI: clindamycin. DAP: daptomycin. ERY: erythromycin. NIT: nitrofurantoin. LIN: linezolid. AMP: ampicillin. OXA+: oxacillin + 2% NaCl. PEN: penicillin. LEV: levofloxacin. CIP: ciprofloxacin. MOX: marbofloxacin. TET: tetracycline. RIF: rifampicin. QUD: quinupristin/dalfopristin. ^a–j^: each superscript in each column signify statistically significant differences (*p*-value ≤ 0.05) in the resistance observed against the various antibiotics investigated. ±: standard error.

**Table 5:** AMR in commensal and infection-causing *Staphylococcus aureus* isolated from healthy cats and cats with an active skin infection.

AB Group	AB	% AMR/AB in Commensal *S. aureus*	% AMR/AB in Infection-Causing *S. aureus*
**Aminoglycosides**	GEN	0 ^a^ (0/6) ± 0	0 ^a^ (0/1) ± 0
**Amphenicols**	CHL	16.7 ^a,b^ (1/6) ± 15.2	0 ^a^ (0/1) ± 0
**Cephalosporins**	CXI	0 ^a^ (0/6) ± 0	0 ^a^ (0/1) ± 0
**Folate Inhibitor Pathway**	TRS	0 ^a^ (0/6) ± 0	0 ^a^ (0/1) ± 0
**Glycopeptides**	VAN	0 ^a^ (0/6) ± 0	100 ^b^ (1/1) ± 0
**Glycylcyclines**	TIG	0 ^a^ (0/6) ± 0	0 ^a^ (0/1) ± 0
**Lincosamides**	CLI	0 ^a^ (0/6) ± 0	0 ^a^ (0/1) ± 0
**Lipopeptides**	DAP	0 ^a^ (0/6) ± 0	0 ^a^ (0/1) ± 0
**Macrolides**	ERY	50 ^b^ (3/6) ± 20.4	100 ^b^ (1/1) ± 0
**Nitrofurans**	NIT	0 ^a^ (0/6) ± 0	100 ^b^ (1/1) ± 0
**Oxazolidinones**	LIN	0 ^a^ (0/6) ± 0	0 ^a^ (0/1) ± 0
**Penicillins**	AMP	16.7 ^a,b^ (1/6) ± 15.2	100 ^b^ (1/1) ± 0
	PEN	16.7 ^a,b^ (1/6) ± 15.2	100 ^b^ (1/1) ± 0
**Quinolones**	LEV	0 ^a^ (0/6) ± 0	100 ^b^ (1/1) ± 0
	CIP	0 ^a^ (0/6) ± 0	100 ^b^ (1/1) ± 0
	MOX	0 ^a^ (0/6) ± 0	100 ^b^ (1/1) ± 0
**Tetracyclines**	TET	0 ^a^ (0/6) ± 0	100 ^b^ (1/1) ± 0
**Ansamycins**	RIF	0 ^a^ (0/6) ± 0	100 ^b^ (1/1) ± 0
**Streptogramins**	QUD	0 ^a^ (0/6) ± 0	0 ^a^ (0/1) ± 0

AB: antibiotic. AMR: antimicrobial resistance. GEN: gentamicin. CHL: chloramphenicol. CXI: cefoxitin. TRA: trimethoprim–sulfamethoxazole. TIG: tigecycline. CLI: clindamycin. DAP: daptomycin. ERY: erythromycin. NIT: nitrofurantoin. LIN: linezolid. AMP: ampicillin. PEN: penicillin. LEV: levofloxacin. CIP: ciprofloxacin. MOX: marbofloxacin. TET: tetracycline. RIF: rifampicin. QUD: quinupristin/dalfopristin. ^a,b^: different superscripts in each column indicate statistically significant differences (*p*-value ≤ 0.05) for the resistance found against the different antibiotics studied. ±: standard error.

**Table 6:** AMR in commensal *Staphylococcus pseudintermedius* isolated from healthy cats.

AB Group	AB	% AMR/AB in Commensal *S. pseudintermedius*
**Aminoglycosides**	GEN	0 ^a^ (0/3) ± 0
**Amphenicols**	CHL	33.3 ^a,b,c^ (1/3) ± 27.2
**Folate inhibitor pathway**	TRS	0 ^a^ (0/3) ± 0
**Glycopeptides**	VAN	0 ^a^ (0/3) ± 0
**Glycylcyclines**	TIG	0 ^a^ (0/3) ± 0
**Lincosamides**	CLI	66.7 ^b,c^ (2/3)
**Lipopeptides**	DAP	33.3 ^a,b,c^ (1/3) ± 27.2
**Macrolides**	ERY	66.7 ^b,c^ (2/3) ± 27.2
**Nitrofurans**	NIT	0 ^a^ (0/3) ± 0
**Oxazolidinones**	LIN	0 ^a^ (0/3) ± 0
**Penicillins**	AMP	33.3 ^a,b^ (1/3) ± 27.2
	OXA+	33.3 ^a,b^ (1/3) ± 27.2
	PEN	100 ^c^ (3/3) ± 0
**Quinolones**	LEV	0 ^a^ (0/3) ± 0
	CIP	33.3 ^a,b^ (1/3) ± 27.2
	MOX	0 ^a^ (0/3) ± 0
**Tetracyclines**	TET	0 ^a^ (0/3) ± 0
**Ansamycins**	RIF	0 ^a^ (0/3) ± 0
**Streptogramins**	QUD	0 ^a^ (0/3) ± 0

AB: antibiotic. AMR: antimicrobial resistance. GEN: gentamicin. CHL: chloramphenicol. TRA: trimethoprim–sulfamethoxazole. TIG: tigecycline. CLI: clindamycin. DAP: daptomycin. ERY: erythromycin. NIT: nitrofurantoin. LIN: linezolid. AMP: ampicillin. OXA+: oxacillin + 2% NaCl. PEN: penicillin. LEV: levofloxacin. CIP: ciprofloxacin. MOX: marbofloxacin. TET: tetracycline. RIF: rifampicin. QUD: quinupristin/dalfopristin. ^a–c^: in each column, different superscripts indicate statistically significant differences (*p*-value ≤ 0.05) for the resistance found against the different antibiotics studied. ±: standard error.

## Data Availability

The data presented in this study are available on request from the corresponding author.

## Supplementary Materials

The following supporting information can be downloaded at: https://www.mdpi.com/article/10.3390/vetsci11020054/s1, Part A: Questionnaire. AMR study in companion animals; Table S1: AMR of all the strains isolated; Table S2: AMR patterns.
