# Supplementary material for: Multidrug-Resistant Commensal and Infection-Causing Staphylococcus spp. Isolated from Companion Animals in the Valencia Region
Source: Vet Sci. 2024 Jan 26;11(2):54. doi: 10.3390/vetsci11020054 (PMC10891909; doi:10.3390/vetsci11020054)
Supplement: Supplementary file 1 [file vetsci-11-00054-s001.zip › Table S2. AMR patterns.pdf]

Table S2. Number of dog and cat *Staphylococcus* spp. strains isolated resistant to the different number of antimicrobials tested and their antimicrobial resistance patterns by antibiotic groups.

N of AB groups	n of dog isolates (%)	n of cat isolates (%)	N of isolates (%)	AMR patterns
**0**	7 (6.2)	15 (20.3)	22 (11.8)	-
	-	2 (2.7)	2 (1.1)	AMPH
	-	1 (1.4)	1 (0.5)	LINCO
	2 (1.8)	4 (5.4)	6 (3.2)	MACR
**1**	5 (4.4)	4 (5.4)	9 (4.8)	PEN
	-	2 (2.7)	2 (1.1)	QUIN
	-	3 (4.1)	3 (1.6)	ANS
	2 (1.8)	1 (1.2)	3 (1.6)	TET
	-	1 (1.4)	1 (0.5)	AMINO-MACR
	-	1 (1.4)	1 (0.5)	AMPH-MACR
	3 (2.7)	-	3 (1.6)	AMPH-OXAZ
	4 (3.5)	-	5 (2.7)	AMPH-PEN
	-	1 (1.4)	1 (0.5)	AMPH-ANS
	1 (0.9)	1 (1.4)	2 (1.1)	AMPH-TET
	1 (0.9)	-	1 (0.5)	CEPHA-PEN
**2**	1 (0.9)	-	1 (0.5)	CEPHA-TET
	-	1 (1.4)	1 (0.5)	GLYC-FOL
	-	1 (1.4)	1 (0.5)	GLICO-MACR
	-	1 (1.4)	1 (0.5)	LINC-PEN
	-	1 (1.4)	1 (0.5)	LINC-MACR
	1 (0.9)	-	1 (0.5)	LINC-OXAZ
	-	1 (1.4)	1 (0.5)	LINC-TET
	-	2 (2.7)	2 (1.1)	MAC-PEN

	-	1 (1.4)	1 (0.5)	OXAZ-QUIN
	2 (1.8)	-	1 (0.5)	OXAZ-PEN
	1 (0.9)	-	2 (1.1)	PEN-MACR
	-	1 (1.4)	1 (0.5)	PEN-QUIN
	1 (0.9)	-	1 (0.5)	PEN-TET
	-	1 (1.4)	1 (0.5)	QUIN-TET
			1 (0.5)	
	-	1 (1.4)	1 (0.5)	AMINO-ANS-LINC
	-	1 (1.4)	1 (0.5)	AMPH-LIPO-PEN
	-	1 (1.4)	1 (0.5)	AMPH-GLICO-LINC
	1 (0.9)	-	1 (0.5)	AMPH-GLYC-OXAZ
	1 (0.9)	-	1 (0.5)	AMPH-LINC-PEN
	-	1 (1.4)	1 (0.5)	AMPH-LINC-TET
	-	1 (1.4)	1 (0.5)	AMPH-QUIN-TET
	1 (0.9)	-	1 (0.5)	STREP-GLYC-LINC
**3**	-	1 (1.4)	1 (0.5)	STREP-PEN-ANS
	2 (1.8)	-	2 (1.1)	MACR-PEN-TET
	-	1 (1.4)	1 (0.5)	NIT-ANS-TET
	1 (0.9)	-	1 (0.5)	GLYC-QUIN-TET
	-	1 (1.2)	1 (0.5)	LINC-MACR-PEN
	-	1 (1.4)	1 (0.5)	LINC-OXAZ-TET
	-	1 (1.4)	1 (0.5)	LINC-PEN-QUIN
	1 (0.9)	-	1 (0.5)	LIPO-MACR-PEN
	1	1 (1.4)	1 (0.5)	LIPO-PEN-ANS
	-	1 (1.4)	1 (0.5)	AMINO-AMPHE- LIPO-ANS
**4**	-	1 (1.4)	1 (0.5)	AMINO-MACR-NITRO-PEN
	1 (0.9)	-	1 (0.5)	AMPH-FOL-MACR-PEN

	1 (0.9)	-	1 (0.5)	AMPH-FOL-OXAZ-TET
	-	1 (1.4)	1 (0.5)	AMPH-GLICO-MACR-PEN
	1 (0.9)	-	1 (0.5)	AMPH-GLYC-CLIN-QUIN
	6 (5.3)	1 (1.4)	7 (3.7)	AMPH-LINC-MACR-PEN
	2 (1.8)	1 (1.4)	3 (1.6)	AMPH-LIPO-MACR-TET
	-	1 (1.4)	1 (0.5)	CEPHA-LINC-LIPO-PEN
	-	1 (1.4)	1 (0.5)	STREP-LINCO-PEN-TET
	-	1 (1.4)	1 (0.5)	FOL-LINC-MACR-TET
	1 (0.9)	-	1 (0.5)	GLYC-QUIN-ANS-TET
	1 (0.9)	-	1 (0.5)	LINC-MACR-OXAZ-PEN
	-	1 (1.4)	1 (0.5)	LINC-MACR-PEN-QUIN
	1 (0.9)	-	1 (0.5)	MACR-CEPHA-PEN-TET
	1 (0.9)	-	1 (0.5)	MACR-OXAZ-PEN-TET
**5**	-	1 (1.4)	1 (0.5)	AMINO-LINC-PEN-QUIN-TET
	2 (1.8)	-	2 (1.1)	AMPH-FOL-CEPHA-PEN-TET
	1 (0.9)	-	1 (0.5)	AMPH-GLYC-LINC-MACR-PEN
	1 (0.9)	-	1 (0.5)	AMPH-GLYC-PEN-QUIN-TET
	1 (0.9)	-	1 (0.5)	AMPH-GLYC-OXAZ-QUIN-TET
	1 (0.9)	-	1 (0.5)	AMPH-LINC-MACR-CEPHA-PEN
	-	2 (2.7)	2 (1.1)	AMPH-LINC-MACR-PEN-QUIN
	3 (2.7)	-	3 (0.5)	AMPH-LINC-MACR-PEN-TET
	1 (0.9)	-	1 (0.5)	AMPH-LINC-MACR-QUIN-TET
	1 (0.9)	-	1 (0.5)	AMPH-MACR-PEN-ANS-TET
	1 (0.9)	-	1 (0.5)	GLYC- CEPHA-MACR-PEN-QUIN
	1 (0.9)	-	1 (0.5)	GLYC-CEPHA-PEN-QUIN-TET
	1 (0.9)	-	1 (0.5)	GLYC-FOL-PEN-QUIN-TET
	-	1 (1.4)	1 (0.5)	LINC-MACR-PEN-QUIN-TET

**6**	1 (0.9)	-	1 (0.5)	AMINO-AMPH-GLYC-MACR-PEN-QUIN
	1 (0.9)	-	1 (0.5)	AMINO-AMPH-MACR-PEN-QUIN-TET
	1 (0.9)	-	1 (0.5)	AMINO-FOL-MACR-PEN-QUIN-TET
	1 (0.9)	-	1 (0.5)	AMPH-CEPHA-GLYC-PEN-QUIN-TET
	-	1 (1.4)	1 (0.5)	AMPH-CEPHA-LINC-MACR-PEN-QUIN
	1 (0.9)	-	1 (0.5)	AMPH-STREP-LINC-MACR-PEN-QUIN
	1 (0.9)	-	1 (0.5)	AMPH-GLICOP-LINC-MACR-OXAZ-PEN
	2 (1.8)	1 (1.4)	3 (2.7)	AMPH-LINC-MACR-PEN-QUIN-TET
	1 (0.9)	-	1 (0.5)	AMPH-LINC-MACR-OXAZ-PEN-ANS
	1 (0.9)	-	1 (0.5)	GLYC-LINC-OXAZ-PEN-QUIN-TET
	1 (0.9)	-	1 (0.5)	STREP- CEPHA-GLYC-LINC -PEN-QUIN
	1 (0.9)	-	1 (0.5)	STREP-GLYC-LINC-MACR-OXAZ-PEN
**7**	1 (0.9)	-	1 (0.5)	AMINO-STREP-LINC-MACR-OXAZ-PEN-QUIN
	1 (0.9)	-	1 (0.5)	AMINO-GLYC-MACR-PEN-QUIN-ANS-TET
	2 (1.8)	-	2 (1.1)	AMINO-GLYC-LINC-MACR-PEN-QUIN-TET
	1 (0.9)	-	1 (0.5)	AMINO-GLYC-MACR-PEN-QUIN-ANS-TET
	1 (0.9)	-	1 (0.5)	AMINO-GLICOP-MACR-OXAZ-PEN-ANS-TET
	3 (2.7)	-	3 (2.7)	AMPH-CEPHA-GLYC-LINC-MACR-PEN-QUIN
	2 (1.8)	-	2 (1.1)	AMPH-FOL-GLICOP-LINC-MACR-PEN-QUIN
	2 (1.8)	-	2 (1.1)	AMPH-GLYC-LINC-MACR-PEN-QUIN-TET
	1 (0.9)	-	1 (0.5)	AMPH-GLYC-MACR-OXAZ-PEN-QUIN-TET
	1 (0.9)	-	1 (0.5)	AMPH-GLYC-OXAZ-PEN-QUIN-ANS
	1 (0.9)	-	1 (0.5)	GLYC-CEPHA-LINC-MACR-PEN-QUIN-TET
	-	1 (1.4)	1 (0.5)	GLICO-MACR-NITRO-PEN-QUIN-ANS-TET
**8**	3 (1.8)	-	3 (2.7)	AMINO-AMPH-FOL-LINC-MACR-PEN-QUIN-TET
	1 (0.9)	-	1(0.5)	AMINO-AMPH-GLYC-LINC-MACR-PEN-QUIN-TET
	1 (0.9)	-	1 (0.5)	AMINO-AMPH-LINC-MACR-NITRO-OXAZ-PEN-TET

	1 (0.9)	-	1 (0.5)	AMPH-CEPHA-GLYC-LINCO-MACR-NITRO-PEN-QUIN
	1 (0.9)	-	1 (0.5)	AMPH-STREP-LINC-MACR-CEPHA-PEN-QUIN-TET
	1 (0.9)	-	1 (0.5)	AMPH-GLYC-FOL-LINC-MACR-PEN-QUIN-TET
	1 (0.9)	-	1 (0.5)	STREP-GLYC-LINC-MACR-CEPHA-PEN-QUIN-TET
**9**	1 (0.9)	-	1 (0.5)	AMINO-AMPH-CEPHA-STREP-GLYC-LINC-MACR-PEN
	1 (0.9)	-	1 (0.5)	AMINO-AMPH-FOL-GLYC-LINC-MACR-NITRO-PEN-QUIN
	1 (0.9)	-	1 (0.5)	AMPH-CEPHA-FOL-GLYC-LINC-MACR-PEN-QUIN-TET
	1 (0.9)	-	1 (0.5)	AMPH-CEPHA-GLYC-GLICOP-LINC-MACR-OXAZ-PEN-QUIN
**10**	1 (0.9)	-	1 (0.5)	AMINO-AMPH-CEPHA-FOL-GLYC-LINC-MACR-PEN-QUIN-TET
	-	1 (1.4)	1 (0.5)	AMINO-CEPHA-LINC-LIPO-MACR-NITRO-PEN-QUIN-ANS-TET
	-	1 (1.4)	1 (0.5)	AMPH-FOL-LINC-LIPO-MACR-NIT-OXAZ-QUIN-ANS-TET
**11**	1 (0.9)	-	1(0.5)	AMINO-AMPH-CEPHA-FOL-GLYC-LINC-MACR-OXAZ-PEN-QUIN-TET
	1 (0.9)	-	1 (0.5)	AMINO-CEPHA-STREP-GLYC-LINC-MACR-OXAZ-PEN-QUIN-ANS-TET

N: total number. n: number. AB: antibiotics. AMR: antimicrobial resistance. AMINO: aminoglycosides. AMPH: amphenicols. CEPHA: cephalosporins. FOL: folate inhibitor pathwas. GLICOP: Glycopeptides. GLYC: glycylcylines. LINC: lincosamides. LIPO: lipopeptides. MACR: macrolides. NITRO: nitrofurans. OXAZ: oxazolidinones. PEN: penicillins. QUIN: quinolones. ANS: Ansamycin. STREP: steptogramins. TET: tetracyclynes.
